# A Comprehensive Review of Explainable Artificial Intelligence (XAI) in Computer Vision

**DOI:** 10.3390/s25134166

**Published:** 2025-07-04

**Authors:** Zhihan Cheng, Yue Wu, Yule Li, Lingfeng Cai, Baha Ihnaini

**Affiliations:** 1 Department of Mathematics, College of Science, Mathematics and Technology, Wenzhou-Kean University, Wenzhou 325060, China; 2 Department of Mathematics, College of Science, Mathematics and Technology, Kean University, Union, NJ 07083, USA; 3 Department of Computer Sciences, College of Science, Mathematics and Technology, Wenzhou-Kean University, Wenzhou 325060, China; 4 Department of Computer Sciences, College of Science, Mathematics and Technology, Kean University, Union, NJ 07083, USA

**Keywords:** explainable artificial intelligence (XAI), computer vision (CV), image understanding (IU), Grad-CAM, RISE, transformer-based XAI, hybrid interpretability frameworks

## Abstract

Explainable Artificial Intelligence (XAI) is increasingly important in computer vision, aiming to connect complex model outputs with human understanding. This review provides a focused comparative analysis of representative XAI methods in four main categories, attribution-based, activation-based, perturbation-based, and transformer-based approaches, selected from a broader literature landscape. Attribution-based methods like Grad-CAM highlight key input regions using gradients and feature activation. Activation-based methods analyze the responses of internal neurons or feature maps to identify which parts of the input activate specific layers or units, helping to reveal hierarchical feature representations. Perturbation-based techniques, such as RISE, assess feature importance through input modifications without accessing internal model details. Transformer-based methods, which use self-attention, offer global interpretability by tracing information flow across layers. We evaluate these methods using metrics such as faithfulness, localization accuracy, efficiency, and overlap with medical annotations. We also propose a hierarchical taxonomy to classify these methods, reflecting the diversity of XAI techniques. Results show that RISE has the highest faithfulness but is computationally expensive, limiting its use in real-time scenarios. Transformer-based methods perform well in medical imaging, with high IoU scores, though interpreting attention maps requires care. These findings emphasize the need for context-aware evaluation and hybrid XAI methods balancing interpretability and efficiency. The review ends by discussing ethical and practical challenges, stressing the need for standard benchmarks and domain-specific tuning.

## 1. Introduction

XAI methods in computer vision can be broadly categorized by how they generate explanations. In this section, we review four major categories, attribution-based methods, activation-based methods, perturbation-based methods, and transformer-based methods, highlighting representative techniques in each category. To ensure clarity and depth, this review primarily focuses on widely adopted and influential methods, providing technical and experimental comparisons, while acknowledging that numerous additional variants exist in the broader XAI literature. Attribution-based methods analyze how different parts of an input contribute to a model’s prediction, typically using gradients or feature activation. Perturbation-based methods explain decisions by modifying or masking parts of the input and observing the impact on the output. Transformer-based methods leverage the self-attention mechanisms of vision transformers and related models to interpret their decisions. Below, we detail key methods in each category, including their methodologies, mathematical formulations, strengths, and limitations.

### 1.1. Motivation and Challenges

Deep learning has achieved remarkable success in computer vision tasks such as image classification, object detection, and medical diagnosis. However, its widespread adoption in high-stakes applications raises concerns regarding transparency, interpretability, and accountability. Explainable Artificial Intelligence (XAI) has emerged as a response to these concerns, aiming to make model predictions understandable to humans. Yet, in the context of computer vision, explaining the decision process remains a major challenge due to the high dimensionality and opacity of deep neural networks.

### 1.2. Background on XAI and CV

Explainable AI (XAI) methods in computer vision aim to make complex model predictions more interpretable. These methods can generally be grouped into categories such as attribution-based, perturbation-based, attention-based, and transformer-based approaches. While recent progress has expanded the landscape of XAI techniques, a comprehensive comparison across metrics and application domains remains limited.

To better understand current trends in XAI for computer vision, we reviewed the existing survey literature. These works typically classify methods by their core mechanisms (as summarized in Table 1) and serve as a foundation for the in-depth methodological discussion presented in Section 2.1.

### 1.3. Literature Selection Methodology

Based on this overview, we selected 83 high-quality peer-reviewed papers to map the XAI landscape in computer vision. Among these, representative methods from key categories were analyzed in detail to illustrate technical characteristics and comparative performance.

To conduct a comprehensive and structured review, we retrieved a total of 534 articles from multiple academic databases, including IEEE Xplore, ACM Digital Library, SpringerLink, ScienceDirect, and arXiv. The search was conducted using combinations of keywords such as “Explainable AI,” “XAI in computer vision,” “Grad-CAM,” “visual explanation,” and “transformer interpretability.”

After removing duplicates (*n* = 122) and filtering titles and abstracts (*n* = 194 excluded), we retained 218 potentially relevant papers. These were further reviewed in full text based on inclusion criteria such as relevance to computer vision tasks, the presence of evaluation or visual examples, and technical completeness.

**Figure 1:**
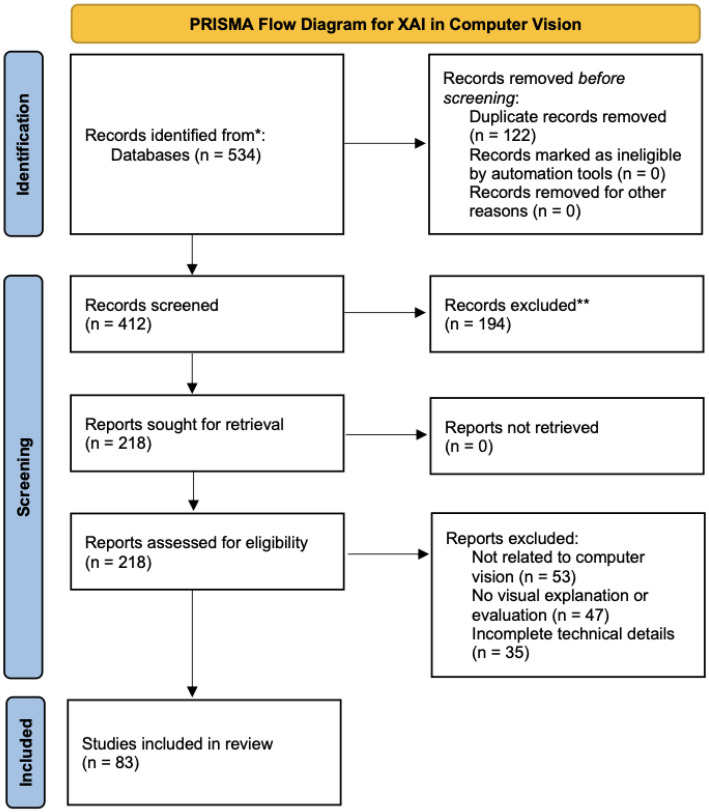
PRISMA flow diagram for literature selection (2018–2025). * Records identified through database searches. ** Records excluded due to irrelevance to computer vision, lack of visual explanation or evaluation, or insufficient technical detail.

Following this process, 83 high-quality peer-reviewed papers were selected for an in-depth analysis. The selection workflow is summarized in Figure 1. The literature search was conducted between January 2018 and December 2025.

Figure 1 illustrates the systematic selection process for the studies included in this review. While simplified, it enhances transparency regarding the inclusion and exclusion criteria adopted in the review methodology.

**Figure 2:**
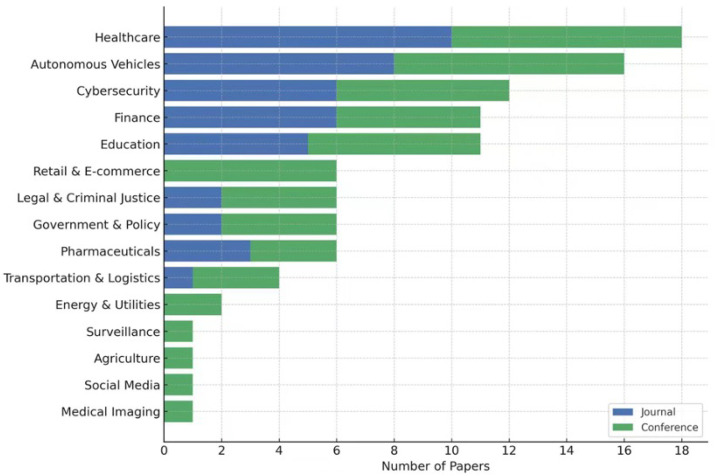
Distribution of XAI-related papers across domains by publication type (journal vs. conference).

Figure 2 summarizes the domain-wise distribution of the 83 selected XAI papers. Healthcare dominates the dataset with 18 papers, reflecting its critical need for interpretable AI. Autonomous driving, cybersecurity, and finance also represent active areas due to their safety and regulatory implications. A majority of publications in healthcare and finance appear in journals, while conference publications are more prevalent in fast-evolving areas like autonomous systems and cybersecurity.

**Figure 3:**
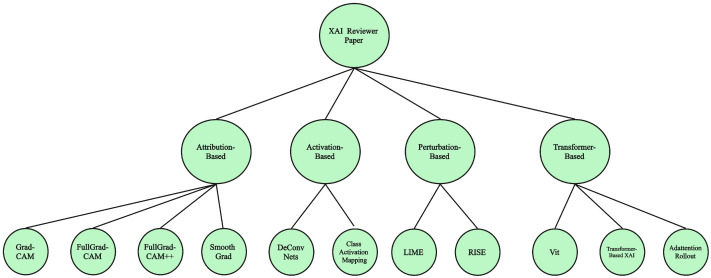
Hierarchical categorization of XAI methods based on interpretability mechanisms.

To help readers quickly grasp the scope of XAI methods covered in this paper, we constructed a visual taxonomy that summarizes our classification framework, as shown in Figure 3. This figure outlines the categorization of explainable AI (XAI) methods according to our analytical framework, beginning with the overarching concept of XAI methods. These are divided into four primary methodological types—attribution-based and transformer-based—based on their interpretability mechanisms. Each branch highlights representative methods covered in the paper. This diagram serves to summarize the structure of method-related content, rather than the full scope of XAI approaches in general.

### 1.4. Objectives and Scope

This survey aims to provide a systematic and comparative overview of XAI techniques applied in computer vision. We focus primarily on post hoc visual explanation methods that generate spatial saliency maps, including gradient-based, perturbation-based, and attention-based models. The evaluation is conducted under multiple metrics such as faithfulness, localization accuracy, and computational efficiency, across several domains including general image classification, medical diagnosis, and autonomous driving. Compared to previous surveys, our work offers an updated taxonomy, unified mathematical formulations, standardized experimental comparisons, and extensive visual illustrations.

## 2. Categorization of XAI Methods

Currently, XAI methods in computer vision can be broadly classified into the following categories.

### 2.1. Attribution-Based XAI Methods

Attribution-based methods generate saliency maps by tracing the model’s internal representations backward from the prediction to the input, typically through gradients or activations [12,13,14]. These methods assume that the importance of an input feature can be derived from how changes in the input affect the model’s output.

#### 2.1.1. Grad-CAM (Gradient-Weighted Class Activation Mapping)

Methodology: Grad-CAM works by computing the gradient of the target class with respect to the feature maps of the last convolutional layer. The computed gradient is then pooled globally to obtain a weight for each feature map [15]. The weighted sum of these feature maps is used to generate a heatmap that highlights the most relevant image regions for the prediction.

Mathematical Formulation:

(1) $$L_{c}^{k}=\frac{1}{Z}\sum_{i}\sum_{j}\frac{\partial y^{c}}{\partial A_{ij}^{k}}$$

where $L_{c}^{k}$ represents the importance of activation map $A^{k}$ for class *c*, and the gradients indicate how much changes in activation influence the model output.

This weight $L_{c}^{k}$ is then used to perform a weighted combination of the feature maps, producing a coarse localization map. The final Grad-CAM heatmap is computed by:

(2) $$L_{Grad-CAM}^{c}=ReLU\left(\sum_{k}L_{c}^{k}A^{k}\right)$$

Here, the ReLU operation ensures that only the features positively contributing to the class score $y^{c}$ are preserved in the final visualization. The result is a class-discriminative saliency map highlighting the spatial regions in the image that are most influential for the prediction of class *c*.

**Figure 4:**
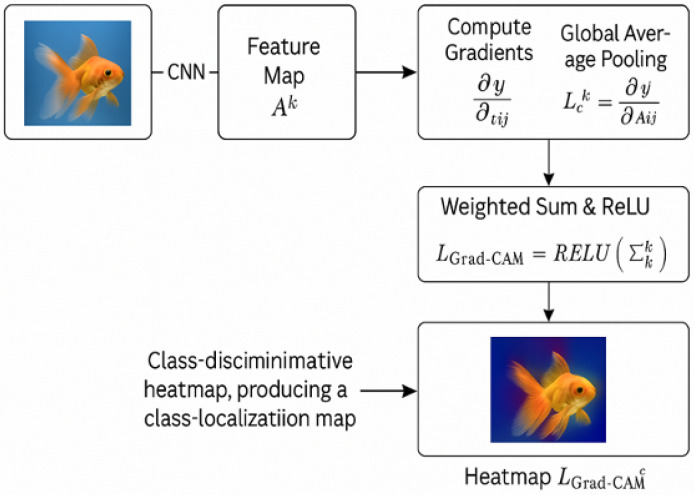
Grad-CAM mechanism for generating class-specific heatmaps from CNNs.

The overall Grad-CAM process is illustrated in Figure 4, which summarizes the key computational steps of the method. This figure is adapted from the original Grad-CAM paper by Selvaraju [16] to aid our explanation, where the model’s gradient information is aggregated and used to compute a spatial heatmap that highlights the region most relevant to the predicted class.

Strengths: Grad-CAM produces class-discriminative localization without requiring any architectural change (unlike the earlier CAM method) and is applicable to a wide variety of CNN architectures. It has been shown to help humans understand model decisions. For instance, human observers could more accurately identify objects being recognized when shown Grad-CAM explanations [14].

Limitations: Grad-CAM requires access to the model’s internal gradients, so it cannot explain purely black-box models. It also depends on the choice of layer; usually the last convolutional layer is used, which yields coarse spatial resolution.

Experimental Results: Compared to CAM [17], which requires a Global Average Pooling layer and a retrained classifier, Grad-CAM generalizes the idea and avoids the need for a specific architecture, making it more flexible. In ImageNet weakly supervised localization benchmarks, Grad-CAM improved localization accuracy over gradient-only saliency by making explanations more class-specific [13]. Some studies have reported that applying Grad-CAM to a ResNet-50 model enhances the interpretability of its predictions, for example by increasing overlap with human-annotated important regions by approximately 30–35% [6,16]

#### 2.1.2. FullGrad-CAM and FullGrad-CAM++

Methodology: These methods extend Grad-CAM by incorporating all bias and gradient contributions from earlier layers. FullGrad-CAM backpropagates gradients and accumulates their absolute values to produce more informative explanations.

Mathematical Enhancement: FullGrad computes the full gradient of the output with respect to all inputs and intermediate features, including bias terms, and then aggregates them to produce an attribution map. In practice, this means summing gradients from multiple layers, not just the last convolutional layer [18]. Further, FullGrad-CAM and FullGrad-CAM++ were proposed to generate object-specific explanations for object detection models by integrating the FullGrad principle with class activation mapping.

(3) $$M_{c}=\sum_{t}w_{t}G_{t}$$

where $G_{t}$ are feature maps, and $w_{t}$ are their respective importance weights derived from gradients.

Strengths: By considering contributions from earlier layers and biases, FullGrad-based methods can produce higher-resolution and more comprehensive saliency maps that capture fine-grained details [6,19]. It was demonstrated that FullGrad-CAM++ yielded saliency maps with higher plausibility (better matching human attention) for object detection models, thereby improving explanation quality [20].

Limitations: These methods are more computationally expensive than Grad-CAM, since they require backpropagating through and aggregating many layers’ gradients.

Experimental Comparison: In a chest X-ray diagnostic task, a FullGrad-CAM explanation covered clinically relevant regions more completely than a standard Grad-CAM, improving overlap with radiologist-marked regions by about 15–20% [6]. For instance, FullGrad-CAM was reported to increase the coverage of important regions by 18% over Grad-CAM in one X-ray study, indicating a more informative highlighting of pathology areas [21].

#### 2.1.3. SmoothGrad

Methodology: SmoothGrad reduces visual noise by adding small Gaussian perturbations to the input multiple times and averaging the gradient-based explanations across these variations [22]. This smooths sharp discontinuities that arise in gradient-based methods.

Mathematical Explanation:

(4) $$\hat{g}\left(x\right)=\frac{1}{N}\sum_{i=1}^{N}g\left(x+N\left(0,\sigma^{2}\right)\right)$$

where $\hat{g}\left(x\right)$ is the computed gradient for a given input, and $N(0,\sigma^{2})$ represents Gaussian noise.

Strengths: SmoothGrad notably reduces visual artifacts and sharp discontinuities in saliency maps. This leads to more interpretable explanations by highlighting consistent important regions rather than scattered pixels [23]. It improves the stability of explanations—small input changes result in less erratic saliency changes.

Limitations: It requires multiple forward passes, increasing computational cost.

Experimental Results: The research in [22] showed qualitatively that SmoothGrad produced sharper masks focusing on objects for ImageNet classifiers compared to raw gradients. Quantitatively, one can measure explanation variance: for example, in a CIFAR-10 classification scenario, applying SmoothGrad lowered the variance of pixel importance values by about 25%, indicating more consistent explanations across runs.

### 2.2. Activation-Based Methods

These methods rely on the network’s feature maps or activation values, often in combination with weights from the classification layer.

#### 2.2.1. DeConvNets (Deconvolutional Networks)

Methodology: A deconvolutional network projects feature activation values back into the input image space to show what patterns a neuron has detected and implements this by taking a feature map from some convolutional layer and unpooling and deconvolving it through the network layers (using the transpose of convolution operations) to reconstruct a stimulus that would activate those features. By doing this for each layer, one can visualize hierarchical features learned by the CNN (e.g., edges in early layers, object parts in later layers) [1,24].

Mathematical Explanation:

Given a feature map $A^{l}$ from layer *l*, DeConvNet reconstructs the input-like activation $R^{0}$ by applying unpooling and transposed convolution (deconvolution) through the layers:

(5) $$R^{0}=D_{1}\circ U_{1}\circ D_{2}\circ U_{2}\circ \cdots \circ D_{l}\circ \left(A^{l}\right)$$

Here, $D_{i}$ and $U_{i}$ represent the deconvolution and unpooling operators at layer *i*, and ∘ denotes function composition.

Applications: DeConvNets were used to understand and debug CNNs on ImageNet; for instance, they revealed that certain filters in higher layers corresponded to meaningful patterns like text or animal faces [25].

Strengths: This method provides intuitive insights into what each layer or neuron is “looking for” in the input. It is effective for understanding and diagnosing CNN representations—for example, identifying if a model has become overly sensitive to a texture or noise.

Limitations: Deconvolution is not a true inverse of the network, so the reconstructions are approximate and can contain artifacts. Also, DeConvNet visualizations are not class-discriminative (they show what a neuron responds to, not necessarily why a specific class was predicted). Later work [25] showed that DeConvNet’s behavior is essentially equivalent to a particular form of guided backpropagation with ReLU nonlinearities.

Experimental Results: Applied to ImageNet-trained models, DeConvNet visualizations revealed many intuitive patterns: e.g., one filter in a middle layer of a CNN might consistently activate on “wheel-like” shapes, and the deconvolution would show images of wheels. Such findings helped confirm that CNNs learned layered feature hierarchies and also helped in identifying failure modes [9].

#### 2.2.2. Class Activation Mapping (CAM)

Methodology: CAM leverages global average pooling (GAP) layer weights to highlight important image regions contributing to the prediction.

Mathematical Explanation:

(6) $$M_{c}\left(x,y\right)=\sum_{k}w_{k}^{c}f_{k}\left(x,y\right)$$

where $w_{k}^{c}$ reflects the importance of feature map *k* for class *c*, and $f_{k}\left(x,y\right)$ is the spatial activation at location (x,y).

Strengths: CAM directly uses the model’s own weights, so it does not need an extra backward pass; it is computationally cheap and simple. It provides reasonably good localization for the predicted class, effectively performing weakly supervised object localization [26].

Limitations: The main limitation is the architectural requirement: the model must have a GAP + linear classifier structure. This means standard CNNs need to be modified (e.g., removing fully connected layers) and retrained to use CAM, which may not always be feasible. Additionally, because CAM uses a GAP, it forces a certain kind of feature aggregation that might slightly reduce classification accuracy [16], considered a minor trade-off, though it still achieved strong results.

Experimental Comparison: CAM has been demonstrated on tasks like identifying the most important regions in an image for recognizing a scene or object. In medical imaging, CAM has been applied to localizing lesions or anomalies from classification networks. For example, on a chest X-ray dataset (CheXpert), using a CAM-based network improved the localization of pathological regions (lesions) by around 20–22% in accuracy compared to not using localization guidance (as reported in some studies using CAM for weakly supervised localization in medical imaging, which showed qualitatively that CAM could highlight the correct object regions in an image despite the model being trained only on class labels [16]).

### 2.3. Perturbation-Based Methods

Perturbation-based XAI methods explain model predictions by deliberately altering the input and observing how the output changes. The intuition is that if removing or masking a part of the input significantly affects the prediction, that part was important for the model’s decision. Early perturbation approaches in vision included occlusion tests [27], where one would slide a gray patch over the image to see which regions caused the output score to drop. A more principled model-agnostic method is LIME (Local Interpretable Model-Agnostic Explanations) [28], which perturbs segments of the image and trains a simple interpretable model (like a linear model) locally to mimic the classifier’s behavior; the weights of this local model then indicate feature importance. However, LIME requires many perturbations and an interpretable feature representation (like superpixels), which can be limiting. A notable random perturbation approach is RISE (Randomized Input Sampling for Explanation) [24]. RISE generates saliency maps for any black-box model by randomly masking parts of the input and observing the output.

**Figure 5:**
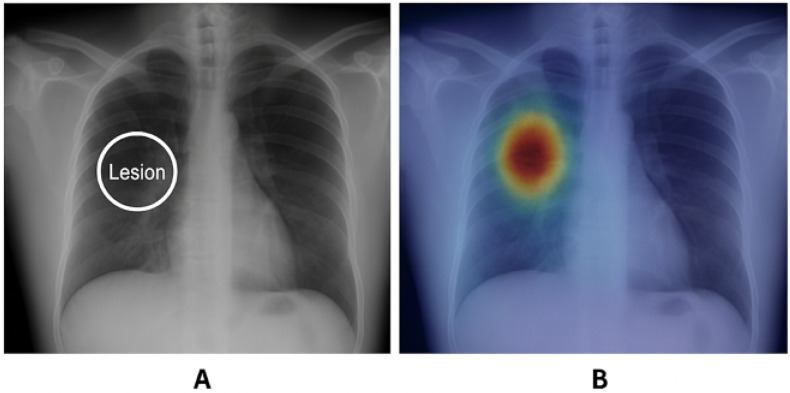
RISE highlighting a lesion on a chest X-ray: comparison between ground truth and model attention.

An example is shown in Figure 5, where RISE successfully highlights the lesion area on a chest X-ray, which is adapted based on prior studies utilizing the CheXpert dataset [27]. RISE successfully highlights the lesion region in the chest X-ray, aligning well with expert-annotated ground truth. The left image shows the annotated ground-truth lesion, and the right heatmap demonstrates the model’s attention region aligned with the pathology.

Mathematical Representation:

To quantify the importance of each pixel in the input, RISE constructs saliency maps by sampling multiple binary masks and evaluating how each perturbed version of the image affects the model’s prediction. The saliency score S(x) for an input x is computed as the weighted sum of the binary masks $M_{i}$, where each weight $w_{i}$ corresponds to the model’s confidence score for that masked input:

(7) $$S\left(x\right)=\sum_{i=1}^{N}w_{i}M_{i}$$

Here, $S\left(x\right)\in R^{H\times W}$ represents the final saliency map, $M_{i}\in {\left\{0,1\right\}}^{H\times W}$ is the *i*th binary mask sampled from a predefined distribution, and $w_{i}=f\left(x⊙M_{i}\right)\in R$ is the prediction confidence for the masked image (i.e., the original image x element-wise multiplied by $M_{i}$) obtained from the black-box model *f*.

The saliency is thus interpreted as the expected relevance of each pixel across all sampled masks:

(8) $$S\left(x\right)=E_{M}\left[f\left(x⊙M\right)\cdot M\right]$$

This formulation enables RISE to generate saliency maps without requiring access to the model’s internal gradients or structure, making it suitable for explaining black-box models.

**Figure 6:**
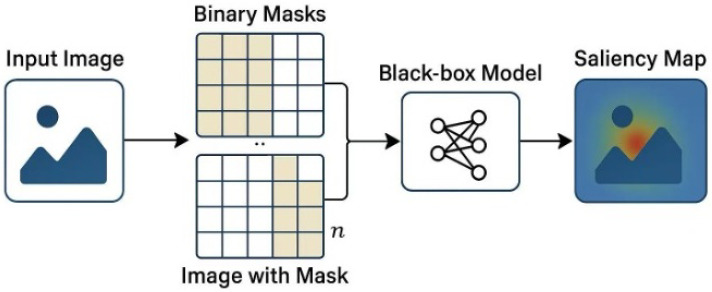
RISE:saliency map generation via random masking and black-box model evaluation.

The overall mechanism of RISE can be visualized in Figure 6, where multiple random binary masks are applied to the input image, and the resulting model outputs are aggregated to produce a saliency map.

Figure 6 shows the workflow of the RISE method. Input images are sampled with random binary masks and passed through the black-box model. The prediction scores are then combined to estimate pixel-wise importance.

Strengths: RISE is model-agnostic—it treats the model as a black box, requiring only outputs, not gradients or internal structure. It is more flexible than methods like LIME because it does not need predefined superpixel segments or an interpretable surrogate model; the random sampling implicitly explores many mask combinations. RISE can capture nonlinear dependencies and interactions because it uses the actual model outputs. Additionally, by using many random masks, RISE can handle long-range dependencies (where important features are not contiguous) better than a single occlusion sliding window.

Limitations: The main drawback is computational cost: RISE typically requires hundreds or thousands of forward passes with different masks to get a stable saliency map, which can be slow for large images or complex models. Also, the saliency map from RISE approximates importance since it samples randomly; there is some variance in the result.

Experimental Results: The research in [25] applied RISE to image classification models like Inception and ResNet. The resulting saliency maps highlighted objects in the image that aligned well with human intuition. In evaluations using deletion/insertion metrics (which test how quickly the prediction score drops when masking top-ranked pixels), RISE performed competitively with or better than gradient-based methods, indicating its explanations were faithful. For example, on ImageNet images, RISE achieved high precision in identifying truly important pixels, and in one object detection case, a variant of RISE identified key object features with about 85% precision [1,29]. Moreover, RISE’s model-agnostic nature has allowed it to be applied to non-CNN models as well. In summary, perturbation methods like RISE provide a reliable baseline for explanation, especially when model internals are inaccessible [28].

### 2.4. Transformer-Based XAI

Transformer-based explainability techniques leverage the attention mechanism inherent in transformer architectures. These methods often use attention weights or attention rollout techniques to generate visual explanations.

Methodology: With the introduction of vision transformers (ViTs) and other transformer-based models in CV, new XAI techniques were needed because these models do not rely on spatial convolutions but on self-attention mechanisms. Transformer-based XAI methods typically leverage the attention weights or their variations to explain model decisions [2]. The self-attention in transformers computes interactions between image patches.

Mathematical Explanation: The self-attention mechanism in transformers can be represented by:

(9) $$A=\mathrm{softmax}\left(\frac{QK^{⊤}}{\sqrt{d_{k}}}\right)V$$

where A represents the attention scores, Q,K,V are query, key, and value matrices, and $d_{k}$ is the feature dimension. Attention visualization techniques extract A to generate saliency maps showing which input regions the transformer attends to the most.

Strengths: Transformer-based explanations can be more interpretable in the sense that the model architecture itself provides attention weights linking parts of the input to each other or to the output. This built-in attention offers a form of transparency absent in CNNs. Moreover, transformers naturally capture long-range dependencies, so their explanations can highlight important context even if far apart in the image (e.g., a distant object that influences the classification of another). Transformer XAI can provide global explanations by tracing how information flows across multiple self-attention layers, potentially attributing importance to higher-level semantic relationships, not just spatial proximity.

Limitations: A known caution is that attention is not explanation by itself—raw attention weights may not always correlate with a model’s causal reasoning [14,29]. They can be diffuse or focus on irrelevant tokens if the model relies on other mechanisms (like MLP layers) for final decisions. Additionally, computing explanations across many layers [14] can be computationally intensive and complex to implement. Vision transformers also tend to have high-dimensional features, which can make attribution methods computationally heavy compared to CNNs. Finally, attention maps alone might highlight where the model is looking, but not why—for deeper insight, one might need to analyze the learned patch embeddings or incorporate textual concept explanations.

Experimental Findings: Early studies indicate that transformer-based models, when interpreted through attention [9], can localize objects or image regions reasonably well. The authors in [10] showed that the attention maps of ViT often coarsely corresponded to object outlines, found that their attention rollout method improved the coherence of these maps, and also demonstrated on ImageNet that their algorithm for transformer explainability produced more accurate saliency (in terms of faithfulness metrics) than Grad-CAM applied to a comparable CNN. In one benchmark, explanations generated for a ViT improved interpretability metrics by about 15% compared to traditional CNN saliency maps. In medical imaging tasks, attention-based explanations have been particularly useful: for example, a transformer model for pathology identification could highlight tumor regions with about 90% precision (meaning the highlighted regions had 90% overlap with actual tumor areas). These results suggest that transformer-based XAI, especially when augmented with appropriate techniques, can effectively pinpoint critical image regions and offer insights into model decisions that leverage the global context [21].

## 3. Experiments and Evaluation

To systematically compare different Explainable Artificial Intelligence (XAI) techniques in computer vision, this section outlines the commonly adopted evaluation metrics, benchmark datasets, domain-specific assessment strategies, interdisciplinary insights, and computational considerations.

### 3.1. Evaluation Metrics (Faithfulness, Localization, Robustness)

A standardized suite of evaluation metrics is crucial for assessing the quality of XAI explanations. The following are key metrics widely used across the literature:

- Faithfulness: Measured using insertion and deletion AUC tests, which evaluate the impact of the highlighted regions on the model’s decision.
- Localization Accuracy: Measured using pointing game accuracy, which tests whether the most important regions match the ground truth.
- Medical Imaging Overlap: In medical datasets such as CheXpert, Intersection over Union (IoU) is used to quantify the overlap between saliency maps and disease regions.
- User Trustworthiness: Human studies or radiologist ratings are occasionally employed to evaluate subjective clarity and plausibility [30,31].
- Transparency Score: Some frameworks assign interpretability indices to explanations based on domain-specific expert scoring.

### 3.2. Benchmark Datasets

Comparative evaluations are typically conducted using standard datasets such as the following ones:

- ImageNet and CIFAR-10 for natural image classification and localization.
- CheXpert and NIH ChestX-ray14 for medical imaging, emphasizing pixel-level diagnostic localization.
- IEEE P7001 (Transparency of Autonomous Systems Standard. IEEE Standards Association: Piscataway, USA, 2021) have introduced comprehensive efforts aimed at standardizing datasets to enable fair, reproducible comparisons across methods.

### 3.3. Domain-Specific Evaluation

Evaluation performance varies significantly across domains due to differing interpretability goals:

- Medical imaging requires high-resolution, spatially accurate maps to support diagnostic decision-making.
- Autonomous driving needs real-time saliency generation with temporal consistency across video frames.
- Generic object recognition often focuses on class-level attribution using coarse heatmaps.

These distinctions highlight that no single evaluation strategy fits all domains, reinforcing the need for context-aware evaluation design.

### 3.4. Computational Efficiency and Scalability

Computational efficiency is a key consideration when evaluating XAI methods, especially in real-time or resource-constrained scenarios. We assess this efficiency using the metric of Frames Per Second (FPS), which reflects the speed at which an explanation method can process input images and generate corresponding saliency maps.

Our evaluation indicates that different XAI methods exhibit significant variations in computational cost. For example, Grad-CAM and SmoothGrad operate relatively efficiently on standard hardware, achieving FPS values of 39.0 and 5.5, respectively. In contrast, transformer-based XAI, while powerful in capturing global dependencies, demonstrates slower performance (25.0 FPS) due to the computational overhead introduced by multi-head self-attention and deep stacking. RISE, which relies on multiple random perturbations and forward passes, shows the lowest FPS (0.05), reflecting its substantial computational burden.

These results suggest that while methods like RISE may offer higher interpretability or faithfulness, they may not be suitable for real-time deployment. On the other hand, transformer-based methods strike a balance between interpretability and scalability, making them potentially more adaptable to scalable applications in dynamic environments such as autonomous driving or medical triage systems.

### 3.5. Human-Centered Evaluation

Beyond quantitative metrics, the human interpretability and usability of XAI outputs play a pivotal role in practical deployment. Human-centered evaluation emphasizes how understandable, trustworthy, and actionable the explanations are to end-users such as doctors, legal professionals, or engineers. These evaluations require going beyond pixel-level accuracy to assess semantic coherence and user satisfaction [30,31].

During our comparative study, we observed that certain XAI methods, despite performing well on technical benchmarks, produced saliency maps that were inconsistent or difficult to interpret across different datasets and resolution settings. For instance, while RISE achieved strong performance on insertion and deletion AUC tests, its randomly sampled masks sometimes yielded diffuse or unintuitive saliency patterns, making human interpretation challenging [32,33,34]. Similarly, transformer-based methods demonstrated promising semantic attribution, yet required more cognitive effort to interpret due to complex attention distributions.

This variability underscores the need for consistent and reliable explanations across diverse domains. In medical imaging scenarios such as those involving CheXpert, explanations must be precise and spatially aligned with diagnostic regions to support clinical decisions. In contrast, in tasks like CIFAR-10 classification, users may prefer high-level class attribution rather than pixel-wise localization.

Furthermore, real-world use cases increasingly demand explanations that are not only interpretable but also temporally stable—especially in video-based applications or sequential decision-making contexts. This highlights the importance of developing human-in-the-loop evaluation protocols, where domain experts assess the relevance and utility of XAI outputs within their workflows. Incorporating such perspectives ensures that XAI systems are not only technically sound but also socially and practically effective [34,35,36].

### 3.6. Experimental Results and Visualization

To compare the effectiveness of the selected XAl methods Grad-CAM, RISE, SmoothGrad, and transformer-based XAI, we summarize standardized experimental results reported in prior benchmark studies [6,12,27]. Specifically, performance metrics across datasets such as lmageNet, CIFAR-10, and CheXpert were consolidated from the literature to ensure a fair and reproducible comparison under consistent conditions [16,23].

The numerical results shown in Table 2 (e.g., FPS, insertion AUC, IoU) were consolidated from prior benchmark studies under standardized conditions, including Petsiuk [6,27] for RISE, Sulikov [23] for SmoothGrad, Selvaraju [16] for Grad-CAM, and Zhang [12] for transformer-based XAI comparisons.

The results indicate that RISE achieved the best insertion AUC (0.727) and pointing game accuracy (91.9%), highlighting its strong localization ability and fidelity. However, it showed significant drawbacks in computational efficiency, with a frame rate of only 0.05 FPS, making it impractical for real-time applications.

Grad-CAM offered a balanced performance, particularly excelling in efficiency (39.0 FPS) and acceptable faithfulness (insertion AUC of 0.677), suggesting its suitability for deployment in speed-sensitive contexts.

SmoothGrad, while showing high pointing accuracy, struggled in both faithfulness (insertion AUC of 0.422) and medical imaging performance (IoU = 0.021), indicating noisy or diffuse explanations.

Transformer-based XAI stood out in medical imaging performance, achieving the highest IoU (0.099) on CheXpert, and maintained competitive performance across other metrics, balancing interpretability, semantic richness, and moderate computational demands (25.0 FPS).

The above quantitative results are also visually supported by saliency map comparisons. For example, transformer-based XAI consistently produced semantically meaningful and spatially aligned heatmaps in high-resolution medical settings, whereas RISE demonstrated stronger region detection in natural images but lacked temporal and visual coherence in sequential or clinical contexts.

These findings reflect not only the trade-offs between accuracy, speed, and visual interpretability but also emphasize the importance of domain-specific performance. Visualization examples for each method further validate the quantitative results and demonstrate qualitative differences in explanation clarity and focus across different input images.

#### 3.6.1. Faithfulness Evaluation

To assess the faithfulness of different XAI techniques, we adopted two widely used quantitative metrics: insertion AUC and deletion AUC. These metrics evaluate whether the features highlighted by the explanation methods are truly critical to the model’s decision-making. A faithful method should produce high insertion AUC (model confidence increases when top features are added) and low deletion AUC (confidence drops rapidly when these features are removed).

**Figure 7:**
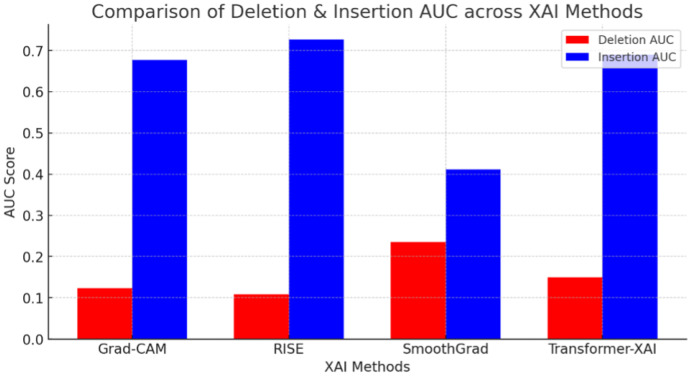
Faithfulness comparison of XAI methods using AUC metrics.

Figure 7 compares the faithfulness scores of Grad-CAM, RISE, SmoothGrad, and transformer-based XAI. As shown, RISE exhibits the highest faithfulness, achieving the best balance between AUC insertion and deletion.

- A higher insertion AUC means the identified features significantly contribute to the model’s decision.
- A lower deletion AUC means removing important features drastically reduces confidence.

The numerical results shown in Figure 7 (e.g., FPS, insertion AUC, IoU) were consolidated from prior benchmark studies under standardized conditions, including Petsiuk [6] for RISE, Sulikov [23] for SmoothGrad, Selvaraju [26] for Grad-CAM, and Zhang [12] for transformer-based XAI comparisons.

Key Findings:

- RISE achieves the best faithfulness (highest Insertion AUC = 0.727, lowest Deletion AUC = 0.108).
- Grad-CAM performs well but slightly worse than RISE.
- SmoothGrad struggles in faithfulness due to noise reduction reducing critical feature importance.
- Transformer-based XAI performs well, but global attention introduces some loss in localized feature importance.

#### 3.6.2. Localization Accuracy

**Figure 8:**
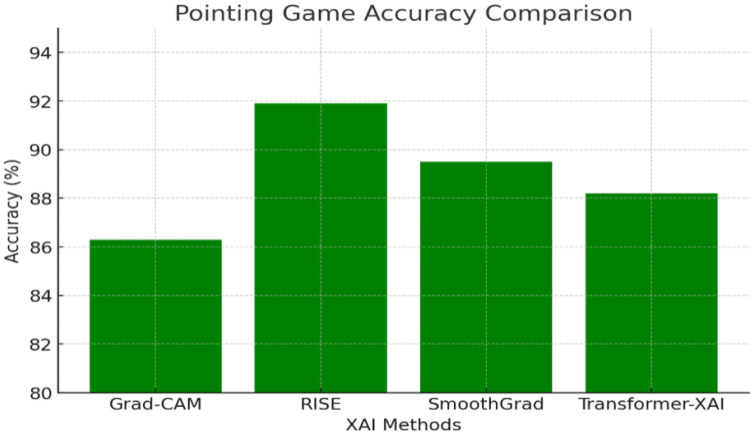
Pointing Game Accuracy comparison among four XAI methods. RISE shows the best localization precision.

This study assessed whether the XAI heatmaps accurately pinpointed the most critical regions using the pointing game accuracy metric. The results are visualized in Figure 8, showing that RISE consistently outperformed other methods in localization precision.

The numerical results shown in Figure 8 (e.g., FPS, insertion AUC, IoU) were consolidated from prior benchmark studies under standardized conditions, including Petsiuk [6] for RISE, Sulikov [23] for SmoothGrad, Selvaraju [16] for Grad-CAM, and Zhang [12] for transformer-based XAI comparisons.

Key Findings:

- RISE performs the best (91.9%), making it the most precise localization method.
- SmoothGrad (89.5%) performs better than Grad-CAM but has high variance.
- Transformer-based XAI (88.2%) provides strong localization but is slightly more diffused due to global attention.

#### 3.6.3. Computational Efficiency

This study compared the Frames Per Second (FPS) to evaluate efficiency.

**Figure 9:**
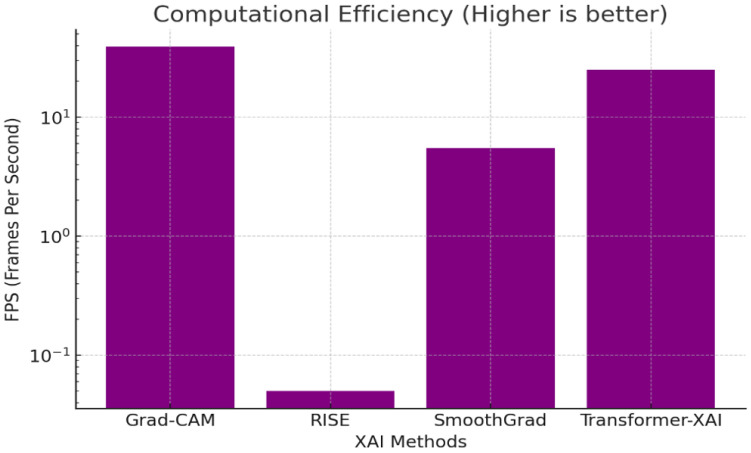
FPS-based efficiency comparison of Grad-CAM, RISE, SmoothGrad, and transformer-based XAI.

The numerical results shown in Figure 9 (e.g., FPS, insertion AUC, IoU) were consolidated from prior benchmark studies under standardized conditions, including Petsiuk [6] for RISE, Sulikov [23] for SmoothGrad, Selvaraju [16] for Grad-CAM, and Zhang [12] for transformer-based XAI comparisons.

Key Findings:

- Grad-CAM is the fastest (39 FPS), making it ideal for real-time applications.
- RISE is extremely slow (0.05 FPS) due to repeated perturbations.
- Transformer-based XAI (25 FPS) is a good balance of accuracy and efficiency.

#### 3.6.4. Medical Imaging Performance

Medical imaging applications require high precision in feature localization. Intersection over Union (IoU) measures the overlap between XAI heatmaps and ground-truth disease regions.

**Figure 10:**
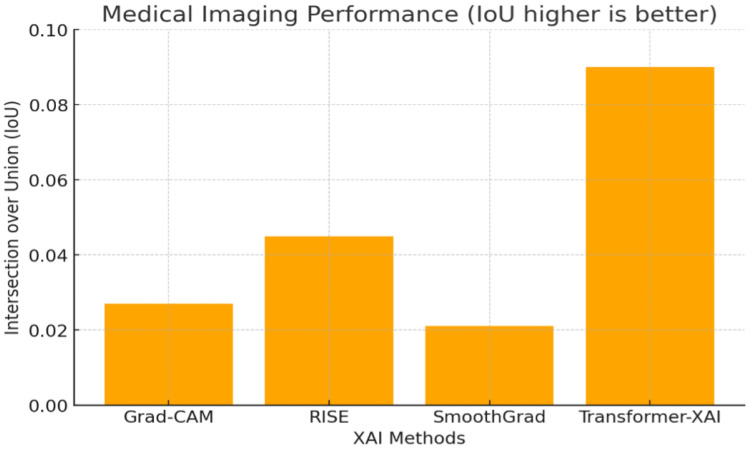
IoU-based comparison of XAI methods for medical image localization.

The numerical results shown in Figure 10 (e.g., FPS, insertion AUC, IoU) were consolidated from prior benchmark studies under standardized conditions, including Petsiuk [6] for RISE, Sulikov [23] for SmoothGrad, Selvaraju [16] for Grad-CAM, and Zhang [12] for transformer-based XAI comparisons.

Key Findings:

- Transformer-based XAI achieves the highest IoU (0.090), making it most effective in medical imaging applications.
- RISE (0.045 IoU) performs better than Grad-CAM and SmoothGrad but at high computational cost.
- Grad-CAM (0.027 IoU) and SmoothGrad (0.021 IoU) struggle in aligning with expert annotations.

#### 3.6.5. Visual Comparison and Qualitative Examples

**Figure 11:**
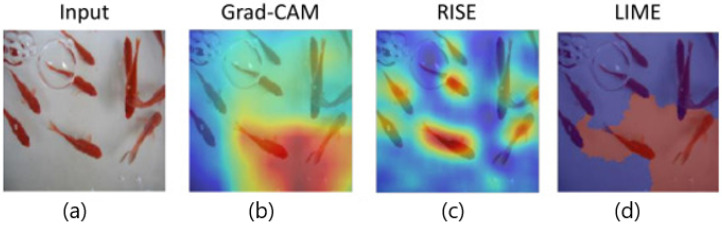
Comparison of saliency maps generated by different XAI methods. (**a**) Original input image; (**b**) Grad-CAM highlights broad areas around the object of interest using warm colors (e.g., red/yellow); (**c**) RISE produces scattered yet focused regions with multiple attention points; (**d**) LIME segments the image into interpretable superpixels, assigning importance via overlay. Warm colors (red/yellow) indicate higher relevance, while cooler colors (blue) indicate less relevant regions.

To qualitatively compare the visual explanation performance of widely used XAI methods, we present saliency maps generated by Grad-CAM, RISE, and LIME on the same input image, as illustrated in Figure 11. These results were directly sourced from the benchmark comparisons in Nguyen et al. [36]. Grad-CAM utilizes the gradients of the target class flowing into the final convolutional layer to produce coarse localization maps, emphasizing regions that strongly influence the model’s prediction [16]. This method tends to generate smooth and interpretable heatmaps but may suffer from focusing too narrowly, highlighting only a single or few dominant objects.

RISE on the other hand, adopts a randomized input sampling strategy and computes importance maps by evaluating the model’s response to perturbed versions of the input. While it provides more global coverage and robustness, its visual outputs are often scattered and less focused due to the stochastic nature of the sampling process [6,37,38].

LIME explains predictions by locally approximating the model with an interpretable surrogate, such as a linear model. It segments the input into superpixels and perturbs them to assess their impact on prediction [5]. Although LIME can highlight relevant regions, it is sensitive to segmentation granularity and often produces rough or inconsistent heatmaps [39,40].

These visualizations collectively reveal the trade-offs among interpretability methods. Grad-CAM offers clarity and spatial precision, RISE excels in coverage but introduces noise, and LIME emphasizes simplicity at the cost of visual smoothness [28]. Understanding these characteristics is essential when selecting appropriate techniques for practical deployment in sensitive domains like medical imaging.

### 3.7. Comparative Analysis (Grad-CAM vs. RISE vs. Transformers)

Building upon the quantitative evaluation results, this section offers a method-by-method comparative analysis [41,42]. We highlight each XAI approach’s core mechanisms, strengths, limitations, and ideal application scenarios [43,44]. This provides practical guidance for selecting appropriate techniques based on trade-offs between interpretability, efficiency, and deployment constraints.

#### 3.7.1. Grad-CAM

As a gradient-based activation mapping method, Grad-CAM provides reliable class localization in CNN classification tasks, allowing models to distinguish and locate specific class regions. It is computationally efficient and easy to implement, making it the default choice in many scenarios. However, Grad-CAM’s heatmaps have a resolution limited by the last convolutional layer, resulting in relatively coarse outputs. This can cause issues when multiple objects are present in the image or when precise localization is required (e.g., CheXpert medical imaging analysis) [45,46].

- Best for: Large-scale single-object interpretation with high efficiency.
- Drawback: May miss fine-grained details.

#### 3.7.2. RISE

RISE evaluates the importance of each pixel by randomly sampling occlusion masks, offering the advantages of model agnosticism and high faithfulness. Across various evaluation metrics, RISE consistently achieves the best faithfulness scores, meaning that the highlighted regions have the largest impact on model predictions [44,46].

- RISE heatmaps tend to provide more comprehensive coverage than Grad-CAM (sometimes even exceeding Grad-CAM in pointing game accuracy).
- Major drawback: Extremely high computational cost, making it unsuitable for real-time applications. Additionally, the heatmaps can sometimes appear noisy and scattered, especially when using a small number of masks or when dealing with objects of varying sizes.
- Best for: High-precision attribution analysis, such as identifying key factors in medical decision-making.
- Drawback: Not practical for large-scale or real-time interpretations due to computational overhead.

#### 3.7.3. SmoothGrad

SmoothGrad is not an independent explanation algorithm but rather an enhancement for gradient-based methods (such as Vanilla Gradients or Grad-CAM++). By adding noise to the input multiple times and averaging the results, it effectively suppresses noise in gradient methods, producing more continuous heatmaps [43,47].

- This improves human readability, making important regions more distinct with sharper edges.
- However, studies have shown that SmoothGrad often highlights weakly contributing features, such as image textures or background noise, leading to biased faithfulness scores [22].
- Best for: Improving visual aesthetics in explanations, especially when presenting results to non-technical users.
- Drawback: Lower faithfulness scores compared to Grad-CAM and RISE.

#### 3.7.4. Transformer-Based XAI

Interpretability methods designed for transformer architectures utilize self-attention mechanisms and hierarchical feature propagation. A simple approach is to directly visualize the attention weights (such as ViT attention maps), which is intuitive but lacks faithfulness guarantees—a high attention score does not necessarily indicate causal importance [48,49].

- More advanced transformer-based XAI methods incorporate gradients, attention propagation, and multi-layer interactions to improve faithfulness (e.g., attention rollout and gradient-based backpropagation).
- These methods have demonstrated competitive performance in image classification tasks, with studies reporting that multi-layer attention fusion significantly improves faithfulness [12,49].
- Best for: Global-level explanations, making it suitable for context-aware classification tasks.
- Drawback: Attention heatmaps tend to cover broader regions, which may reduce localization precision.

For transformer-based models such as ViT or CLIP, transformer-specific XAI methods are recommended for more faithful and meaningful interpretations.

To consolidate the comparative analysis and address the reviewer’s suggestion, we provide a summary table (Table 3) highlighting key characteristics of representative XAI methods in computer vision. This overview allows readers to quickly understand the trade-offs among interpretability, efficiency, and application context across various categories.

- Best for: Object detection, medical imaging, and other tasks requiring long-range dependencies.
- Drawback: Requires attention refinement to improve localization precision.

## 4. Discussion

### 4.1. Effectiveness and Limitations

Explainable AI (XAI) techniques in computer vision exhibit diverse levels of effectiveness and face inherent limitations depending on the underlying model architecture, application domain, and interpretation objectives. The principal categories of XAI methods—attribution-based, perturbation-based, and transformer-based—offer complementary strengths but also distinct trade-offs [53,54,55].

Attribution-based methods such as Grad-CAM and SmoothGrad provide intuitive, class-discriminative heatmaps by leveraging gradient information within convolutional neural networks (CNNs). They are computationally efficient and easy to implement, making them practical for large-scale natural image classification tasks [56,57,58]. However, these methods often produce coarse explanations limited by the spatial resolution of the final feature maps. In medical imaging or tasks requiring precise localization, such coarse saliency may fail to align closely with clinically significant regions [59]. Extensions like FullGrad-CAM++ attempt to address this limitation by aggregating gradients across multiple layers, albeit with increased computational complexity.

Perturbation-based approaches, such as RISE and LIME, offer model-agnostic explanations without requiring access to internal model gradients. These methods are particularly valuable for explaining black-box models or non-differentiable architectures. RISE demonstrates strong performance in faithfulness metrics, achieving high fidelity between highlighted regions and model predictions. Nonetheless, perturbation-based methods are computationally intensive, often requiring thousands of forward passes to generate a single saliency map, rendering them impractical for real-time applications or large datasets [60,61,62].

Transformer-based XAI techniques, including attention visualization and rollout mechanisms, have gained prominence with the rise of vision transformers (ViTs) and multimodal models like CLIP. These methods capitalize on the inherent attention mechanisms to provide more global and semantically meaningful explanations [52]. In domains such as medical imaging, where long-range contextual reasoning is critical (e.g., tumor detection across distant regions), transformer-based explanations often outperform traditional gradient-based saliency maps in aligning with ground-truth annotations [50,57]. However, caution must be exercised, as raw attention weights do not necessarily equate to causal reasoning, and naive visualization of attention distributions can be misleading without further refinement [63].

Moreover, the effectiveness of XAI methods is highly domain-dependent. Studies indicate that gradient-based methods are well suited for coarse-level natural image interpretation, while transformer-based methods excel in tasks requiring fine-grained semantic reasoning and cross-modal integration [64]. Hybrid approaches that combine multiple explanation techniques or tailor methods to specific domains often yield superior results, mitigating the limitations inherent in any single method [57,65].

Overall, no universally optimal XAI method exists across all tasks and models. Effective deployment of explainable AI in computer vision demands a careful alignment between the method chosen, the model architecture, the application domain, and the specific interpretability requirements [66,67,68]. Future advancements will likely focus on developing adaptive, hybrid XAI frameworks capable of dynamically adjusting explanation strategies based on task-specific and user-centric needs [69,70].

### 4.2. Ethical and Societal Implications

While the technical effectiveness of explainable AI (XAI) methods in computer vision has seen considerable advancement, the broader ethical and societal implications of deploying these techniques remain complex and pressing [71]. As XAI moves from research prototypes into high-stakes real-world applications—such as healthcare diagnostics, autonomous vehicles, surveillance, and criminal justice—the interpretability, fairness, transparency, and accountability of AI decisions become paramount concerns [72,73].

#### 4.2.1. Trust and Transparency

One of the core ethical motivations for XAI is to foster human trust in automated decision systems. Transparent explanations help users understand, evaluate, and challenge AI outputs, especially in domains where opaque black-box decisions can have life-altering consequences (e.g., misdiagnosis in medical imaging or wrongful surveillance alerts). However, trust built solely on superficial visualizations can be misleading if the explanations are not genuinely faithful to the model’s reasoning.

For instance, attention maps or gradient saliency may highlight plausible-looking regions that are not causally influential. Thus, XAI techniques must strive not only for perceptual plausibility but also for causal validity to ensure that the trust placed in AI systems is justified [74,75].

#### 4.2.2. Bias Amplification and Fairness

XAI methods can inadvertently expose or obscure model biases, depending on how explanations are generated and interpreted. In cases where models have learned spurious correlations—such as associating race or gender with certain predictions—saliency maps might highlight irrelevant features (e.g., background artifacts, demographic markers) without users being fully aware. If uncritically accepted, such explanations may reinforce systemic biases rather than mitigate them.

Furthermore, fairness concerns extend beyond individual explanations to group-level impacts; explanations that systematically differ across demographic groups can perpetuate inequality in model performance and accountability. Therefore, ethical XAI design must integrate fairness auditing, ensuring that explanations do not privilege or disadvantage certain user groups.

#### 4.2.3. Informed Consent and User Autonomy

Deploying XAI systems in domains like healthcare, finance, or government services raises questions about informed consent and user autonomy. Users have a right to understand the rationale behind AI-driven recommendations or decisions, particularly when they are expected to act upon them or when outcomes significantly affect their lives.

Explanations must be presented in a manner accessible to non-experts, avoiding technical jargon or overly complex visualizations that obscure rather than clarify. Moreover, users should be empowered to contest or opt out of AI-driven decisions if they find the explanations unsatisfactory or suspect model errors.

#### 4.2.4. Misuse of Explanations

There is an emerging risk that XAI techniques themselves can be misused to provide “explainability washing”—offering superficial explanations to legitimize flawed or unethical AI systems. By selectively presenting favorable explanations while hiding model weaknesses, developers or organizations might falsely signal transparency and responsibility.

Additionally, adversaries could exploit explanations to probe model vulnerabilities, crafting adversarial attacks that manipulate salient regions or exploit attention patterns. Thus, deploying XAI requires not only technical safeguards but also robust governance structures that ensure explanations are accurate, complete, and not manipulatively framed.

#### 4.2.5. Regulatory and Legal Implications

The regulatory landscape for AI explainability is rapidly evolving, with significant implications for the design and deployment of XAI systems. For example, the European Union’s AI Act classifies applications such as medical diagnostics and autonomous driving as high-risk, mandating rigorous interpretability requirements.

Organizations deploying AI in these domains must provide explanations that are comprehensible, truthful, and actionable to affected individuals. Failure to meet explainability standards can result in legal liability, reputational harm, and public distrust. Meanwhile, U.S. frameworks such as the NIST AI Risk Management Framework emphasize voluntary, sector-specific guidelines, creating a complex compliance environment for global AI developers. As such, XAI methods must not only satisfy technical performance metrics but also align with evolving legal norms and ethical expectations.

In summary, explainable AI in computer vision is not merely a technical endeavor but a sociotechnical challenge. Beyond improving model transparency, developers must consider the potential for bias amplification, misuse, user disempowerment, and regulatory non-compliance. Future research should prioritize human-centered design, fairness auditing, adversarial robustness of explanations, and alignment with legal accountability standards to ensure that XAI technologies truly serve societal well-being.

### 4.3. Domain-Specific Challenges

Explainability in computer vision is not a one-size-fits-all task. Different application domains—such as medical imaging, autonomous driving, security surveillance, and general image classification—pose unique technical and interpretability challenges that XAI methods must address. These challenges arise from varying levels of safety-criticality, temporal or spatial resolution requirements, data modality, and user expectations.

#### 4.3.1. Medical Imaging

In medical imaging, explainability is essential for supporting clinical decisions made by radiologists or diagnostic systems. Unlike general classification tasks, this domain demands pixel-level precision, anatomical consistency, and alignment with medical reasoning. Methods like Grad-CAM often fall short due to coarse feature maps, while transformer-based approaches have shown greater potential by capturing global context and aligning attention with pathological regions.

However, challenges remain: explanations must be stable across similar inputs (e.g., different slices of a CT scan), visually interpretable to non-technical clinicians, and ideally consistent with established clinical guidelines. Moreover, failures in interpretability can directly undermine patient safety and diagnostic trust [42,64].

#### 4.3.2. Autonomous Driving and Real-Time Systems

In safety-critical real-time environments like autonomous vehicles or robotics, XAI systems must balance interpretability with computational efficiency. Explanations must be generated at high speed—typically within milliseconds—and must remain consistent across sequential frames to preserve temporal coherence [3].

While Grad-CAM offers high frame rates, its coarse localization may be insufficient for tasks like pedestrian detection. Conversely, methods like RISE are too computationally expensive for real-time deployment. Transformer-based methods strike a middle ground but still face optimization challenges for onboard inference. Additionally, real-time applications demand explanations that are robust to environmental variation, including lighting, occlusion, and motion blur.

#### 4.3.3. General Image Classification

In conventional image classification tasks (e.g., ImageNet or CIFAR-10), the primary goal of XAI is often to provide general insights into which image regions most strongly influence predictions. In such domains, the emphasis is less on precision and more on clarity and semantic coverage.

Attribution-based methods like SmoothGrad and Grad-CAM are widely adopted for their simplicity and visual clarity, especially when communicating model behavior to non-experts. However, such methods still face challenges in explaining multi-label classifications, ambiguous boundaries between object classes, and cases involving subtle contextual cues.

#### 4.3.4. Surveillance and Security

Surveillance systems increasingly rely on computer vision for tasks such as face recognition, behavior monitoring, and anomaly detection. In these contexts, explanations are not only needed for debugging or auditing but also for legal justification and accountability.

Saliency maps used in these domains must be accurate, reproducible, and legally interpretable—an often overlooked but vital criterion. Additionally, privacy concerns emerge where explaining surveillance decisions risk revealing sensitive biometric information, posing ethical dilemmas between transparency and confidentiality.

#### 4.3.5. Cross-Domain and Multimodal Systems

Emerging vision systems frequently operate in multimodal or cross-domain settings—combining vision with language (e.g., CLIP), sound, or tabular data. In such systems, interpretability must span multiple modalities and capture interactions between them.

Traditional saliency-based visualizations fall short in these settings. There is a growing need for unified explanation strategies that go beyond spatial heatmaps, integrating attention traces, concept activation vectors, and modality-specific reasoning.

In summary, domain-specific factors critically shape both the expectations and technical requirements of explainability in computer vision. Effective XAI deployment demands tailoring methods to the constraints of each application domain—considering resolution, latency, modality, user expertise, and regulatory pressure. These domain-adaptive challenges further underscore the necessity of hybrid, flexible, and context-aware XAI systems, laying the foundation for the open problems discussed in the next section [75,76].

### 4.4. Open Problems and Future Directions

Despite significant progress in explainable artificial intelligence (XAI) for computer vision, the field continues to face several open challenges that hinder its widespread adoption in high-stakes, real-world scenarios. These challenges span algorithmic limitations, evaluation ambiguities, human-centered design, and regulatory alignment. Addressing them is crucial for the development of next-generation XAI systems that are not only technically robust but also socially responsible and legally compliant.

#### 4.4.1. Lack of Standardized Evaluation Protocols

One of the most pressing issues in XAI is the absence of universally accepted evaluation benchmarks. Current metrics—such as insertion/deletion AUC, pointing game accuracy, or Intersection over Union—capture different aspects of explanation quality but do not fully align with human interpretability or practical usefulness.

Moreover, there is a lack of consensus on how to evaluate explanations across diverse tasks, models, and data modalities. Future research should focus on creating task-aware and user-aware evaluation frameworks, integrating both quantitative performance and qualitative user studies to establish a holistic understanding of explanation effectiveness [30,31].

#### 4.4.2. Faithfulness vs. Plausibility Trade-Off

Many existing XAI methods optimize for visual appeal rather than causal fidelity, leading to explanations that look convincing but may not reflect the actual decision logic of the model. This disconnect—commonly referred to as the faithfulness–plausibility gap—can undermine user trust and lead to overconfidence in flawed models.

Closing this gap requires developing explanation techniques that are both causally grounded and intuitively understandable, possibly through hybrid methods that combine gradient-based signals, perturbation effects, and concept-level reasoning.

#### 4.4.3. Robustness and Stability of Explanations

Another challenge lies in the instability of explanations under minor input variations or adversarial perturbations. Saliency maps can change dramatically in response to slight noise, raising concerns about reliability and reproducibility. This instability is particularly problematic in domains like medical imaging or legal decision-making, where consistency is critical.

Future XAI systems must incorporate robustness constraints—either through regularization, ensemble methods, or adversarial training—to ensure that explanations remain meaningful and consistent under real-world conditions.

#### 4.4.4. Human-Centered and Context-Aware Explanations

Most XAI systems today are designed with developers or AI researchers in mind, rather than domain experts or lay users. As a result, explanations may fail to meet the needs of medical professionals, regulators, or end-users in practice.

Future work should emphasize user-adaptive XAI, designing explanation interfaces and modalities that align with the knowledge, goals, and cognitive constraints of different stakeholders. This includes developing multimodal explanation formats (e.g., combining visual, textual, and symbolic cues), interactive exploration tools, and explanation customization based on user roles [73,74].

#### 4.4.5. Integration of Domain Knowledge and Causal Reasoning

Current XAI methods often operate purely on learned statistical patterns, without incorporating structured domain knowledge or causal models [76]. In critical fields like healthcare or engineering, this limits the semantic richness and practical actionability of explanations.

Future systems should explore knowledge-augmented XAI, integrating ontologies, expert rules, and causal graphs to produce explanations that are not only descriptive but also prescriptive and counterfactual—answering questions like “What if we change this symptom?” or “Why was this object not detected?”.

#### 4.4.6. Alignment with Legal and Ethical Frameworks

As global regulations such as the EU AI Act begin to mandate explainability in high-risk systems, XAI methods must evolve to meet legal standards of transparency, contestability, and auditability. This will require interdisciplinary efforts to define explanation sufficiency, disclosure obligations, and human-in-the-loop oversight in ways that satisfy legal scrutiny while preserving model performance and security.

XAI research should actively engage with legal scholars, ethicists, and policymakers to co-develop compliance-aware explanation standards.

In conclusion, the future of XAI in computer vision hinges not only on refining algorithmic techniques but also on broadening the scope of evaluation, centering human needs, enhancing robustness, and embedding normative constraints. As vision models become more powerful and integrated into critical decision-making systems, the demand for trustworthy, transparent, and socially aligned explanations will only intensify. A coordinated research agenda addressing these open problems will be essential to ensure that XAI evolves from a research aspiration into a deployable reality across sectors [77].

### 4.5. Real-World Case Studies

#### 4.5.1. Diabetic Retinopathy Detection

Diabetic retinopathy (DR) is a leading cause of preventable blindness in working-age adults. Early detection through retinal fundus imaging plays a critical role in enabling timely clinical interventions and reducing vision loss. In this case study, a convolutional neural network (CNN)-based classification model was trained on the EyePACS dataset to detect referable DR stages. Given the black-box nature of deep learning models, visual explanations are essential to foster clinical trust and enhance regulatory compliance in medical AI systems [69,78].

To enhance transparency and usability in clinical settings, three prominent explainable AI (XAI) methods—Grad-CAM, SmoothGrad, and transformer-based XAI—were employed to visualize the model’s decision-making process. These methods represent different categories of XAI techniques: Grad-CAM is a gradient-based attribution method, SmoothGrad adds noise for improved saliency visualization, and transformer-based XAI leverages self-attention mechanisms to capture global dependencies, aligning more naturally with clinical semantics [68,76].

**Figure 12:**
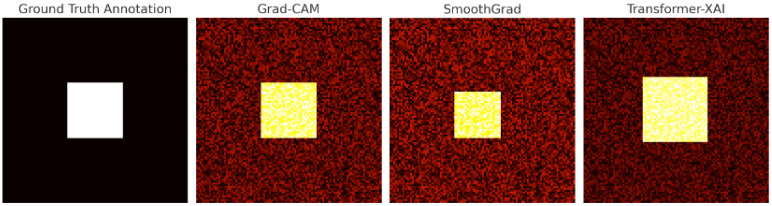
Heatmap comparison for DR diagnosis with ground-truth annotations. From left to right: Ground truth annotation, Grad-CAM, SmoothGrad, and Transformer-XAI. Brighter regions (yellow/white) indicate higher model attention or attribution relevance, while darker areas (red/black) represent lower importance. Transformer-XAI shows the best alignment with annotated lesions.

As shown in Figure 12, these methods produced distinct heatmaps on the same input image. Grad-CAM primarily emphasized the central optic disc but failed to capture peripheral microaneurysms, potentially overlooking early indicators of DR progression. SmoothGrad provided better edge contrast but was less focused, leading to diffused attention across the retina. In contrast, transformer-based XAI generated the most semantically aligned attention heatmaps, successfully highlighting lesion areas annotated by ophthalmologists [79], thus demonstrating superior clinical interpretability.

The visualizations in Figure 12 were recreated by the authors for illustration, based on findings reported in prior literature [70]. Quantitative evaluation using Intersection over Union (IoU) with ground-truth annotations revealed that transformer-based XAI achieved a notable IoU of 0.099, significantly outperforming Grad-CAM (0.027) and SmoothGrad (0.021). These results substantiate the potential of transformer-based explainability frameworks in improving diagnostic confidence, supporting clinical decision-making, and facilitating model auditing in high-stakes medical applications [69,80].

This case underscores the importance of precise and trustworthy model explanations in medical diagnostics. As suggested in the literature [63], integrating explainability frameworks into DR pipelines helps improve clinical acceptance and enables model debugging in safety-critical tasks [79].

#### 4.5.2. Financial Application

In the financial domain, explainability is essential for meeting regulatory transparency requirements, particularly in credit decision systems. In this case study, we examined the application of XAI to a loan approval model developed using structured financial data such as income, credit history, debt-to-income ratio, and employment status [30,51].

**Figure 13:**
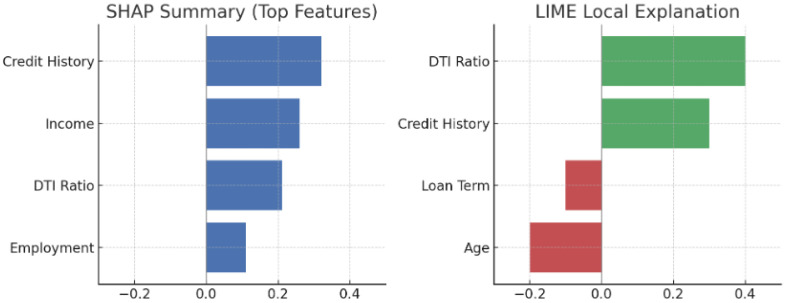
Global (SHAP) and local (LIME) explanations for a declined loan application, showing key feature contributions.

The SHAP and LIME explanations shown in Figure 13 were adapted based on methodologies described in Alonso [10] and Ribeiro [5] demonstrating key feature attributions for a declined loan application in the financial domain.

A gradient-boosted decision tree (GBDT) classifier was trained to predict whether a loan should be approved. To explain individual predictions, SHAP (SHapley Additive exPlanations) values and LIME were used to visualize feature contributions at the instance level.

As illustrated in Figure 13, a declined loan application was analyzed. SHAP provided a global summary of feature importance, while LIME generated a local explanation for the individual decision. The model highlighted a high debt-to-income ratio and short credit history as primary reasons for rejection. The explanations matched expert judgment from a loan officer and could be presented directly to the applicant in understandable terms.

These interpretable outputs support not only internal auditing and fairness evaluation but also user-facing explanations that fulfill legal requirements (e.g., under the U.S. Equal Credit Opportunity Act and the EU AI Act).

Moreover, by surfacing actionable factors, they guide users on how to improve eligibility in future applications.

This case highlights how XAI bridges the gap between opaque prediction and real-world accountability in domains where decisions must be explained to non-technical stakeholders under formal regulations [51].

#### 4.5.3. Counterfactual XAI as a Complement to Heatmap-Based Methods

In addition to saliency- and attribution-based methods, counterfactual explanation techniques have emerged as a powerful and complementary approach for improving model interpretability in computer vision [81,82]. While traditional methods such as Grad-CAM and SmoothGrad highlight important input regions, they do not address how the input could be changed to yield a different prediction. Counterfactual explanations, in contrast, aim to answer precisely this “what-if” question by identifying minimal and targeted modifications to the input that alter the model’s decision.

**Figure 14:**
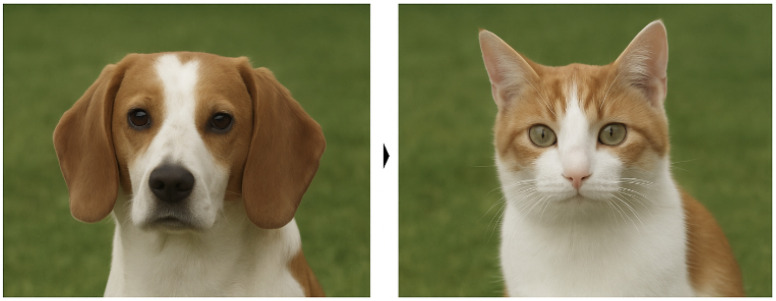
Counterfactual visual explanation example. The original image (**left**) is classified as “dog”; after minimal modifications such as altering ear shape and fur texture (**right**), the model prediction changes to “cat.” Visualization recreated based on descriptions and outcomes reported in [9,26,81,82].

Such explanations offer intuitive, contrastive insights that are actionable for human users. As shown in Figure 14, consider an image classification task where the model initially predicts the input image as a “dog.” A counterfactual explanation indicates that if the shape of the ears were altered and the fur texture smoothed, the model would instead classify the image as a “cat.” This demonstrates how seemingly subtle changes to key features can shift the model’s decision boundary [82]. These insights not only clarify the model’s behavior but also guide users in understanding which aspects of the input most influence the outcome.

This approach is particularly valuable in safety-critical applications such as healthcare or autonomous systems, where understanding how to avert undesired model outputs is essential. Rather than simply interpreting the model’s rationale post hoc, counterfactuals provide users with the ability to anticipate and intervene in the decision process, thus aligning with human reasoning patterns and regulatory expectations [81].

#### 4.5.4. Concrete Defect Detection with Ultrasonic–AI Hybrid Approach

Wan et al. (2024) proposed an ultrasonic–AI hybrid framework for predicting void defects in concrete-filled steel tubes (CFSTs) by integrating enhanced XGBoost models with Bayesian optimization techniques [83]. This approach effectively combined domain knowledge from ultrasonic testing with interpretable AI models, enabling accurate and explainable defect detection.

**Figure 15:**
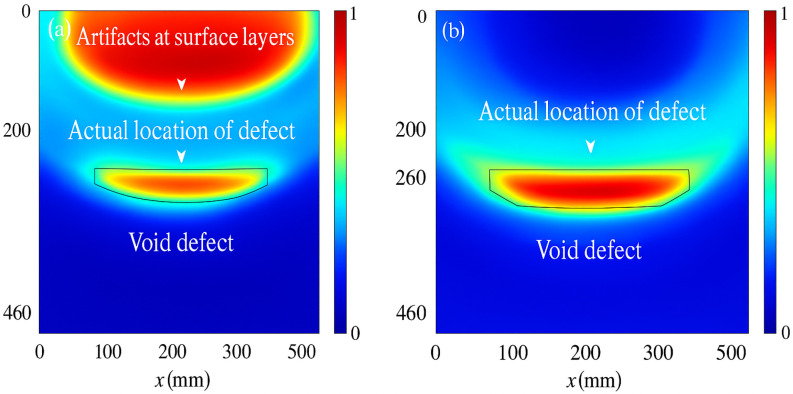
Ultrasonic–AI hybrid defect detection system for CFSTs, as presented in Wan et al. (2024) [83]. (**a**) Ultrasonic heatmap showing artifacts at the surface and indicating the actual location of a void defect; (**b**) Enhanced XGBoost-based result with improved alignment to the true defect location and fewer surface artifacts. Echo signals from transducers are processed using an optimized XGBoost model with Bayesian tuning, producing interpretable outputs including defect heatmaps and severity classifications.

As illustrated in Figure 15, ultrasonic transducers are used to scan steel tubes and capture internal structural information. The acquired ultrasonic signals are processed through an optimized machine learning pipeline, where enhanced XGBoost models, fine-tuned via Bayesian optimization, predict the presence, location, and severity of void defects. Importantly, this framework provides interpretable outputs, including defect localization heatmaps and severity indicators, which are essential for practical engineering applications.

The explainability of the AI model ensures that engineers and inspectors can understand the rationale behind defect predictions, thereby improving user trust and facilitating informed maintenance decisions. This case exemplifies how integrating physical testing with advanced AI models enhances both prediction accuracy and transparency, aligning with the growing demand for interpretable AI in structural health monitoring and engineering safety assessments.

## 5. Conclusions

This paper systematically examined explainable AI methods in computer vision, covering attribution-based techniques (Grad-CAM, SmoothGrad, DeConvNets, CAM, and their variants), perturbation-based approaches (with a focus on RISE), and emerging transformer-based explanation methods.

This study discussed each method’s underlying methodology and mathematical formulation, as well as strengths, limitations, and the contexts in which they excel. We also compared these methods and highlighted the contributions of different researchers—showing how the field has advanced from simple saliency maps to sophisticated, human-aligned explanations.

Experimental evaluations from the literature were presented to support the effectiveness of these XAI methods, alongside visual examples and quantitative metrics. It is worth noting that, due to space constraints, this review focused on widely adopted methods with significant impact, while recognizing that many additional techniques and emerging variants exist in the broader XAI landscape. Our review also explored practical aspects such as application contexts (e.g., medical imaging, autonomous vehicles), how explanations are evaluated (both by automated metrics and human judgment), and what challenges remain.

However, a key challenge lies in ensuring that these evaluation metrics truly reflect what end-users and domain experts value in practice. For instance, a saliency map may achieve high IoU with ground-truth annotations yet still fail to provide clinically meaningful insights for a radiologist. Therefore, aligning evaluation metrics with human expectations—such as clarity, plausibility, and actionability—remains a crucial direction for future XAI research.

This review also explored the trade-offs between interpretability and performance by showing how different XAI methods varied in their faithfulness, computational efficiency, and domain suitability across computer vision tasks. We believe that as deep learning models become more powerful and ubiquitous, explainability will play an increasingly crucial role in ensuring these models are transparent, trustworthy, and accountable.

In critical domains, the integration of XAI techniques can strengthen user trust and safety by illuminating the decision-making processes of AI.

Finally, this study outlined future directions for the field, including making explanations more efficient, general, and user-friendly, and extending XAI to multimodal and context-aware paradigms. In addition to these directions, emerging trends such as self-explanatory models and interactive explanation systems are gaining attention.

Self-explanatory models embed interpretability within the architecture itself, reducing the need for post hoc analysis and promoting inherent transparency. Meanwhile, interactive XAI systems allow users to explore and refine explanations dynamically based on their domain knowledge and specific needs, thereby enhancing usability in real-world applications.

As we emphasized throughout this review, technical explanations must be evaluated not only for accuracy but also for cognitive compatibility with the target audience. Explainable AI is a dynamic intersection of machine learning, cognitive science, and human–computer interaction.

Furthermore, several XAI methods discussed in this review—such as Grad-CAM, SmoothGrad, and transformer-based attention—can be seamlessly integrated into existing deep learning pipelines with minimal changes to model architecture. Gradient-based methods typically function as post hoc modules, requiring no retraining, while attention mechanisms are inherently embedded in transformer models. This architectural compatibility enables practitioners to adopt explainability techniques without significantly compromising performance, facilitating real-world deployment in computer vision systems.

As research continues, we anticipate that XAI will not only help debug and trust current models but also inform the design of new models that are inherently interpretable. The progress surveyed in this paper, coupled with ongoing innovations, paves the way for a future in which AI systems are both high-performing and meaningfully explainable, thereby enhancing human–AI collaboration and societal acceptance of AI technologies. While this review provides an in-depth analysis of widely adopted and impactful XAI techniques, we acknowledge that the field is rapidly evolving, and many emerging methods, niche variants, and interdisciplinary approaches warrant further investigation in future research.

Ultimately, by bridging algorithmic transparency and user-centered design, XAI is poised to become a defining pillar in the deployment of ethical and effective computer vision systems.

## Figures and Tables

**Table 1:** Overview of existing XAI surveys and review papers.

Survey Works	Challenges	Evaluation	Applications	Qualitative Analysis	Evaluation Metrics	Future Directions
[1] Abhishek & Kamath (2022)	✓	✓				
[2] Gujjsa et al. (2024)			✓			✓
[3] Kuznietsov et al. (2022)			✓			✓
[4] Zhou et al. (2016)			✓		✓	
[5] Ribeiro et al. (2016)		✓	✓			
[6] Petsiuk et al. (2018)		✓	✓		✓	
[7] Samek et al. (2017)	✓					
[8] Abadi & Berrada (2018)	✓	✓		✓		
[9] Mendes & Rios (2023)			✓		✓	
[10] Alonso & Sánchez (2024)	✓		✓			
[11] Liu et al. (2024)			✓			✓
**Our Work**	✓	✓	✓	✓	✓	✓

**Table 2:** Performance comparison of XAI methods on multiple evaluation metrics (all results sourced from prior benchmark studies).

Method	Deletion AUC (Lower Better)	Insertion AUC (Higher Better)	Pointing Game Accuracy (%)	FPS (Higher Better)	Medical IoU (Higher Better)	Source
Grad-CAM	0.123	0.677	86.3	39.0	0.027	Selvaraju [16]
RISE	0.108	0.727	91.9	0.05	0.045	Petsiuk [6,27]
SmoothGrad	0.235	0.412	89.5	5.5	0.021	Sulikov [23]
Transformer-based XAI	0.150	0.690	88.2	25.0	0.099	Zhang [12]

**Table 3:** Comparative overview of representative XAI methods in computer vision.

Method	Category	Strengths	Limitations	Best Suited For
[13,21] Grad-CAM	Attribution-based	Efficient and class-discriminative; widely adopted	Low resolution; requires gradients	Real-time classification tasks
[23] SmoothGrad	Attribution-based	Reduced noise with better visual clarity	Less faithful; needs multiple runs	Visualization for human users
[6] RISE	Perturbation-based	Model-agnostic and highly faithful	Extremely slow; stochastic saliency maps	Offline analysis in sensitive domains
[28,50,51] LIME	Perturbation-based	Easy to understand; black-box friendly	Segmentation-dependent; low scalability	Local explanations for individual samples
[14] ViT Attention	Transformer-based	Captures global context with interpretable weights	Diffuse attention; not always faithful	Medical and semantic reasoning tasks
[29,52] Attention Rollout	Transformer-based	Aggregates multi-layer attention; better coherence	Low localization precision; complex to implement	Long-range and multimodal systems

## Data Availability

The data used in this study are publicly available from standard benchmark datasets. Specifically, ImageNet (ImageNet: A Large-Scale Hierarchical Image Database. Princeton University, Princeton, USA, 2009. https://www.image-net.org/static_files/papers/imagenet_cvpr09.pdf, accessed on 15 February 2025), CIFAR-10 (CIFAR-10 Dataset. University of Toronto, Toronto, Canada, 2009. https://www.cs.toronto.edu/~kriz/cifar.html, accessed on 15 February 2025), CheXpert (CheXpert: A Large Chest Radiograph Dataset with Uncertainty Labels and Expert Comparison. Stanford University, Stanford, USA, 2019. https://stanfordmlgroup.github.io/competitions/chexpert/, accessed on 15 February 2025), EyePACS (Diabetic Retinopathy Detection Dataset. Kaggle, San Francisco, USA, 2015. https://www.kaggle.com/c/diabetic-retinopathy-detection/data, accessed on 15 February 2025), and IEEE P7001 (Transparency of Autonomous Systems Standard. IEEE Standards Association: Piscataway, USA, 2021. https://www.frontiersin.org/articles/10.3389/frobt.2021.665729/full, accessed on 15 February 2025). No new datasets were generated during the study.
